# Body image perception of African immigrants in Europe

**DOI:** 10.1186/s12992-016-0184-6

**Published:** 2016-08-23

**Authors:** Stefania Toselli, Natascia Rinaldo, Emanuela Gualdi-Russo

**Affiliations:** 1 Department of Biomedical and Neuromotor Sciences, University of Bologna, Bologna, Italy; 2 Department of Biomedical and Specialty Surgical Sciences, University of Ferrara, Ferrara, Italy

**Keywords:** Body image perception, Dissatisfaction, BMI, Migration, Europe, Health

## Abstract

Nutritional disorders are now spreading worldwide both in developed and developing countries. Body image ideals and dissatisfaction have been linked to a number of poor health outcomes, including nutritional disorders. While previous studies have offered insight into weight status and body image perception of immigrants in North America, very few studies have analysed these aspects in migrants from Africa to Europe. Our review examines the effects of the migration process on beauty ideals and body dissatisfaction in African immigrants in Europe compared to residents in their own countries. The PubMed, PsycINFO and Google Scholar databases were searched for studies published from January 2000 till November 2015. Of the 730 titles identified, 26 met the inclusion criteria and were included in the present review. Among African residents, the body preferences depend on the country of residence and their socio-cultural status. Ethnic groups living in great isolation or with low incomes still have an ancestral idea of beauty, preferring a shapely body. However ethnic groups living in urban areas are moving toward Westernization of beauty ideals, preferring underweight or normal weight bodies. This review highlights that both residents and migrants are at high risk of nutritional disorders due to the adoption of Western beauty ideals. The results suggest that body dissatisfaction and BMI are increasing from Southern Africa to Europe according to a geographical gradient (described for females by Spearman’s coefficient and linear regression, respectively). We emphasize the need for monitoring of the weight and psychological status of immigrants and the development of specific preventive strategies in European countries.

## Background

A wide body of research has described the relationships between health and weight status in different populations and environmental contexts. The categorization of weight status by BMI is a simple way to estimate underweight or overweight for a given height for general assessment of health status in a population. The increasing incidence of overweight and obesity worldwide is now reaching alarming proportions. As an effect of the nutritional transition, these diseases concern both developed and developing countries. Hence the latter are facing a dual burden of malnutrition, with a high prevalence of both underweight and overweight/obesity [1–3].

Body image perception is one of the psycho-social factors that can affect the weight status. As a consequence of a misperception, thin people might overestimate their weight and, conversely, many overweight/obese people are unaware that their body weight is too high. In such cases, as well as in case of dissatisfaction with one’s body image, there is often an association with weight-related behaviors [2, 4].

The relationship between weight status and body self-perception is also influenced by others factors such as cultural and social factors [5–8]. Previous studies in Western countries have reported lower accurate perceptions of overweight in low socio-economic status (SES) groups and this misperception contributes to the persistence of unhealthy lifestyle [9, 10]. This aspect is particularly burdensome in immigrants who, misperceiving their overweight/obesity, do not attempt to lose weight, leading to an increase of these nutritional disorders. Furthermore, people from low-to-medium-income countries who have migrated to high-income countries seem to be more susceptible to overweight and obesity than their local counterparts [11]. In addition, overweight and obesity among immigrants appear to increase significantly with time after migration, with rates approaching or overtaking those of the host population [12]. The process of “acculturation” leads to great changes in the “hosted” group, influencing diet and favouring the adoption of obesogenic behaviours [13, 14].

However, this process is not uniform across all immigrant groups and depends on ethnicity, gender, age at the time of migration and period of residency in the new country [15, 16]. Since socio-cultural factors influence the standards of desirable body weight within cultures, body image perception and body shape preferences are culturally determined [5]. Furthermore, the ideals of beauty may change as a result of immigration, leading immigrant people, especially women, to pursue ideals of thinness. Thus, in immigrants in which the beauty ideals determine an increased level of dissatisfaction, the risk for the development of nutritional disorders increases.

As is the case of immigrants, the comparison of body image perception and body shape preference across populations living in developing countries is complicated by the variety of environmental and cultural conditions. However, the diffusion and subsequent adoption of Western ideals of thinness also in these countries can affect changing body ideals of populations and thus their lifestyle, diet and physical activity patterns. This is related to ‘modernization’, media influence and recent rapid transitions of the economy and urbanization [17–19]. Nevertheless, it is not so clear if the recent preference for slimmer body size is only due to the Westernization process. It is also probably related to the idea that “plumpness” is revered only when it is a rare condition in a context of food scarcity, while it loses desirability when overweight and obesity become more prevalent [17].

Body size and body image perception have mainly been investigated among immigrant groups in the USA [11] and there are few data regarding migrants from Africa to Europe.

In this review we evaluate body dissatisfaction and weight discrepancy among African immigrants in Europe compared to people still living in Africa, tracing a pattern of geographical variation where possible.

Furthermore, specific aims included: to compare body image perception in different population samples from the same country; to compare body image perception between women and men; to consider how weight status interacts with body image perception. The changing self-perception among immigrants entails new challenges in Europe for the development of appropriate strategies aimed at ethnic groups more at risk for nutritional disorders.

## Methods

An extensive search for publications regarding body image perception and body size preferences in different ethnic groups from Africa, both in their countries of origin and after their migration to Europe, was carried out with Web-based search engines (PubMed and PsycINFO). Further searches were carried out in the search engine Google Scholar. In particular, experimental or review articles published from January 2000 to November 2015 were screened by one author (NR) on the basis of titles and abstracts. The inclusion of the full-text studies was then decided by all authors. A combination of the following key words was used for literature identification: “immigration”, “immigrants”, “migrants”, “refugees”, “ethnic minorities”, “body size preferences”, “body dissatisfaction”, “body image dissatisfaction”, “body image”, “weight”, “weight perception”, “Europe”, “Africans”, “North Africa”, “Central Africa”, “Southern Africa”.

**Fig. 1:**
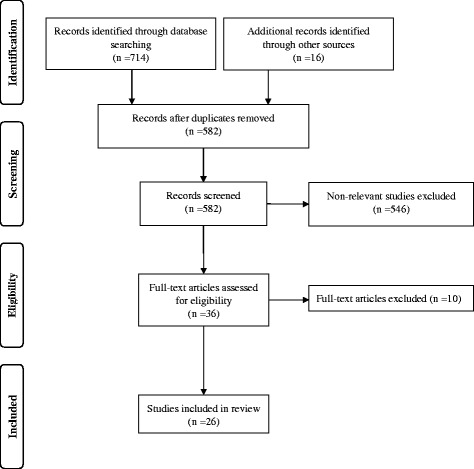
Flowchart of studies for review

In addition, the reference lists of all the selected articles were examined to find other non-indexed papers. In the selection, we took into consideration only cross-sectional studies with samples of healthy subjects aged 15 years or more; longitudinal studies, case reports or studies of subjects in childhood or with concomitant diseases (i.e. diabetes) were excluded. In total, 730 records were found, both through database and references searching, and 582 records were screened after duplicates removal. A number of 36 full-text articles were assessed for eligibility. After the omission of four studies on samples of too young or unhealthy subjects, three studies without body image outcomes and other three studies not focused on African migrants in Europe or on African residents, we included in this review 26 full-text papers (Fig. 1). A summary sheet was developed for extracting data including qualitative or quantitative methodologies to assess body image perception; sample size; age, gender and nationality of participants; host country; body mass index (BMI); prevalence of overweight and obesity (according to WHO cut-off points); actual and ideal figures, level of dissatisfaction. As there are few articles available in the literature on this topic, we considered all of them, irrespective of sample size, for a general listing.

Most of the considered studies classified subjects into weight categories by BMI. In some cases, height and weight were self-reported by the participants. There was wide variability among studies regarding the methods used for the evaluation of body image perception (silhouettes developed by different authors; self-reported questionnaires). In some studies, the Feel minus Ideal Difference index (FID) [20] representing the discrepancy between the actual figure and the ideal figure, was reported as the level of body image dissatisfaction, while in other studies the percentage of satisfaction, or of dissatisfaction (desire to lose/gain weight), was indicated.

An evidence synthesis was carried out by weighted means and pooled standard deviation (SD) of FID and BMI for African residents and immigrants in Europe to check for any trend. Only aggregations of data from studies reporting both BMI and FID for samples ≥ 50 subjects were considered for this purpose. According to the inclusion criteria, only female samples were considered. Linear regression analysis and Spearman R coefficient were carried out to estimate the geographical patterns on the basis of the weighted means of BMI and FID, respectively. For calculation purposes, the geographical areas were identified by numbers 1 (Southern Africa), 2 (Central Africa), 3 (North Africa) and 4 (African immigrants to Europe). Comparisons of mean BMI values between independent samples were carried out by Student’s *t*-test.

The results were statistically significant when p was <0.05. The statistical analysis was carried out using STATISTICA for Windows, Version 11.0 (StatSoft Italia srl, Padua, Italy).

This review was carried out in accordance with the PRISMA guidelines [21].

## Results

### Description of included studies

Twenty-six studies were included in this review on the basis of available data on samples of African origin living in Europe (EU) or still in their own countries (Table 1). Six of them were carried out on body image and body size ideals of Africans who had migrated to EU: three on North African (NA) immigrants in the Netherlands [18, 22, 23], one on NA immigrants in Italy [24] and two on Southern African (SA) immigrants in the UK [25, 26]. For the comparison with their peers still resident in Africa, we utilized studies on Moroccans, Tunisians, Egyptians [17, 22–24, 27–33] and people living in Zimbabwe and South Africa [2, 25, 26, 33–38]. Unfortunately, no study was found on Central African (CA) immigrants in EU. However, for completeness and comparative purposes, we report data for residents in this area [4, 33, 39–43] in order to have a full picture of body image perception and dissatisfaction throughout Africa.

**Table 1:** Studies on body image perception, body size ideal and weight dissatisfaction in African immigrants and residents

Reference (year)	Country of origin/ethnic sample	Eventual host country	Gender: number of subjects	Age (years)	BMI	Actual body image and self-perception	Ideal body image	Dissatisfaction and FID	Methods
NORTH AFRICA									
Nicolau et al. (2008) [22]	Morocco	Amsterdam, The Netherlands	Male: 56	18–30 Mean: 21.6 ± 3.4	BMI: 23.4 ± 2.8 Ow: 19.6 % Ob: 3.6 %	4.1 ± 0.8	Self: 4.2 ± 0.5 Own sex: 4.4 ± 0.7, Opposite sex: 4.2 ± 1.1	Want to be thinner: 21.8 %	7 silhouettes developed by Colllins [49]
			Female: 104	18–30 Mean: 23.4 ± 4.2	BMI: 23.1 ± 4.1 Ow: 24.8 % Ob: 11.5 %	4.6 ± 1.0	Self: 3.9 ± 0.7 Own sex: 4.1 ± 0.6, Opposite sex: 4.2 ± 0.6	Want to be thinner: 57.6 %	7 silhouettes developed by Colllins [49]
Nicolau et al. (2009) [23]; Nicolau et al. (2012) [18]	Morocco	Amsterdam, The Netherlands/ Second generation immigrants	Female: 22	20–59 Mean: 34.5			Preferred silhouettes from 2 to 4. The most attractive is number 4.	A lot of women desire to lose weight	7 silhouettes developed by Colllins [49]
Nicolau et al. (2009) [23]	Morocco (Al Hoceima, Rif region)		Female: 31	16–48 Mean: 25.7			Preferences are between silhouettes 2 to 4. The most attractive is number 4	A lot of women desire to lose weight	7 silhouettes developed by Colllins [49]
Gualdi-Russo et al. *In press* [24]	North Africa (Morocco, Tunisia and Egypt)	Italy	Female: 105	Mean: 36.3 ± 7.8	BMI: 28.4 ± 4.8	5.9 ± 2.1	3.9 ± 1.6	FID: +1.99 ± 2.31	9 silhouettes developed by Thompson and Grey [50]
Gualdi-Russo et al. *In press* [24]	Morocco (Casablanca)		Female: 124	Mean: 39.5 ± 13.1	BMI: 26.4 ± 5.2	5.9 ± 2.0	4.4 ± 1.4	FID: +1.50 ± 1.83	9 silhouettes developed by Thompson and Grey [50]
Gualdi-Russo et al. *In press* [24]	Tunisia (Tunis)		Female: 104	Mean: 28.7 ± 11.5	BMI: 25.5 ± 5.5	5.2 ± 2.4	3.8 ± 1.6	FID: +1.40 ± 2.41	9 silhouettes developed by Thompson and Grey [50]
Lahmam et al. (2008) [30]	Morocco (High Atlas)/ Amazigh people		Male: 165	≥20	BMI: 22.9 ± 3.2 Uw: 6.7 % Nw: 69.01 % Ow: 21.8 % Ob: 2.4 %	False perception: 48.5 %, uw: 46.7 %, ow: 1.8 % Right perception: 51.5 %		Gain weight: 40.0 % Sat: 59.4 % Lose weight: 0.6 %	Self-administered questionnaire
			Female: 271	≥20	BMI: 25.0 ± 4.2 Uw: 3.7 % Nw: 50.2 % Ow: 32.8 % Ob: 13.3 %	False perception: 75.2 %, uw: 74.5 % ow: 0.8 %. Right perception: 24.7 %		Gain weight: 53.1 % Sat: 45.8 % Lose weight: 1.1 %	Self-administered questionnaire
Rguibi et al. (2004) [27]; Rguibi et al. (2006) [28]	Morocco (Laayoun)/Sahraoui women		Female: 249	≥15	BMI: 26.1 ± 5.6 Uw: 2.7 % Nw: 47.1 % Ow: 28.3 % Ob: 21.9 % Ow-Ob: 50.2 %		Ideal body size: 4.88 ± 0.86 Healthy body size: 4.33 ± 0.82	Gain weight: 16.9 % Sat: 79.9 % Lose weight: 3.2 %	9 silhouettes developed by Leandris et al. [51] and self-administered questionnaire
Jafri et al. (2013) [29]	Morocco (Casablanca)		Female: 425	≥18	BMI: 29.9 Ow: 36.2 % Ob: 47.4 %	Right perception: 47 %. Ow-ob underestimate: 36.1 %		Gain weight: 16.7 %	Self-administered questionnaire
Ansari et al. (2013) [31]; Ansari et al. (2014) [32]	Egypt (Assiut)		Male: 1504	Mean: 19.3 ± 1.6	Uw: 7 % Nw: 68 % Ow: 19 % Ob: 6 %	Self-perception: Uw: 17 % Nw: 60 % Ow: 23 %		Sat: 64 % dis: 46 %; no BIC: 74.4 %, mild BIC: 17.3 %, moderate/marked BIC: 8.3 %	Body Shape Questionnaire by Cooper et al. [52]; BIC: Body Image Concern [52]
			Female: 1663	Mean: 18.6 ± 1.2	Uw: 6 % Nw: 62 % Ow: 25 % Ob: 7 %	Self-perception: Uw: 11 % Nw: 56 % Ow: 14 %		Sat: 45 %, dis: 55 %; no BIC: 60 %, mild BIC: 24.2 % moderate/marked BIC: 15.8 %	Body Shape Questionnaire by Cooper et al. [52]; BIC: Body Image Concern [52]
Tlili et al. (2008) [17]	Tunisia (Tunis)		Female: 203	18–52	BMI: 26.1 ± 5.6; Uw: 2.7 % Nw: 47.1 % Ow: 28.3 % Ob: 21.9 % Ow-Ob: 50.2 %			Dis: 62.1 % Lose weight: 47.3 % Gain weight: 14.8 % Ow-ob want to lose weight: 77.9 %	6 photographic silhouettes developed by Bush et al. [44]
Jaeger et al. (2002) [33]	Tunisia (Tunis)		Male and Female: 52	19–23 Mean: 21.4 ± 1.1	BMI: 22 ± 3	3.6	2.8	FID: +0.8	10 silhouettes (self-administered questionnaire)
CENTRAL AFRICA									
Benkeser et al. (2012) [39]	Ghana (Accra Metropolitan Area)		Female: 2814	Mean: 46.28 ± 18.21	BMI: 28.34 ± 6.69	5.05 ± 1.45	4.84 ± 1.45		8 silhouettes developed by Stunkard et al. [53]
Frederick (2008) [4]	Ghana (HO, rural)		Male: 22	Mean: 24.5 ± 8.0			Female ideal body: 4.4 ± 1.4		Contour Drawing Rating Scale (Modified Version; 9 women's silhouettes developed by Thompson and Grey [50])
			Female: 26	Mean: 30.6 ± 12.23		5.1 ± 2.2	4.6 ± 1.4	FID: +0.5 ± 1.7	Contour Drawing Rating Scale (Modified Version; 9 women's silhouettes developed by Thompson and Grey [50])
Siervo et al. (2006) [40]	Gambia (Bakau-Kanifing Municipal Area)		Female: 50	Mean: 18.6 ± 3.4	BMI: 20.6 ± 4.1	4.4 ± 2.3	4.7 ± 1.4	FID: −0.38 ± 2.5	Body Image Assessment for Obesity (18 silhouettes developed by Williamson et al. [54]); 8 silhouettes developed by Stunkard [53]
			Female: 50	Mean: 42.5 ± 5.2	BMI: 30.3 ± 5.2	7.8 ± 3.0	5.0 ± 2.6	FID: +2.8 ± 3.0	Body Image Assessment for Obesity (18 silhouettes developed by Williamson et al. [54]); 8 silhouettes developed by Stunkard [53]
			Male: 50	Mean: 19.3 ± 2.6	BMI: 19.0 ± 2.2	3.7 ± 1.5	4.9 ± 1.6	FID: −1.2 ± 1.9	Body Image Assessment for Obesity (18 silhouettes developed by Williamson et al. [54]); 8 silhouettes developed by Stunkard [53]
			Male: 50	Mean: 42.0 ± 5.3	BMI: 22.3 ± 3.9	5.4 ± 2.6	5.5 ± 2.1	FID: −0.08 ± 1.8	Body Image Assessment for Obesity (18 silhouettes developed by Williamson et al. [54]); 8 silhouettes developed by Stunkard [53]
Holdsworth et al. (2004) [41]	Senegal (Dakar)		Female: 301	20–50	BMI: 25.4 ± 5.6	2.90	2.40		6 photographic silhouettes developed by Bush et al. [44]
Okoro et al. (2014) [42]	Nigeria (Yoruba)		Male: 220	Mean: 42.6 ± 17.2	BMI: 21.7 ± 3.7	4.30 ± 0.99	4.72 ± 1.06		9 silhouettes developed by Becker et al. [55]
			Female: 304	Mean: 44.9 ± 16.7	BMI: 24.6 ± 5.5	4.33 ± 1.17	4.41 ± 1.22		9 silhouettes developed by Becker et al. [55]
Jaeger et al. (2002) [33]	Gabon (Libreville)		Male and Female: 100	19–23 Mean: 19.5 ± 1.3	BMI: 20.8 ± 2.8	4.4	4.2	FID: +0.2	10 silhouettes (self-administered questionnaire)
Jaeger et al. (2002) [33]	Ghana (Techiman)		Male and Female: 58	19–23 Mean: 19.3 ± 1.3	BMI: 22.4 ± 4.6	3.9	4.2	FID: −0.3	10 silhouettes (self-administered questionnaire)
Ettarh et al. (2013) [43]	Kenya (Korogocho and Viwandani slums of Nairobi)		Male: 2669	≥18 Mean: 42	Uw: 9.8 % Nw: 72.9 % Ow: 15.0 % Ob: 2.3 %	Self-perception: Uw: 13.2 % Nw: 52.7 % Ow: 20.8 % Ob: 13.4 %	Ideal body size: Uw: 6.1 %, Nw: 41.3 % Ow: 32.0 % Ob: 20.6 %		18 silhouettes developed by Williamson et al. (1989) [54]
			Female: 2265	≥18 Mean: 42	Uw: 5.1 % Nw: 51.5 % Ow: 27.9 % Ob: 15.5 %	Self-perception: Uw: 14.2 % Nw: 50.5 % Ow: 22.2 % Ob: 13.2 %	Ideal body size: Uw: 7.1 % Nw: 53.4 % Ow: 24.8 % Ob: 14.8 %		18 silhouettes developed by Williamson et al. (1989) [54]
SOUTHERN AFRICA									
Swami et al. (2012) [25]	Zimbabwe	UK (London)	Female: 138	18–49 Mean: 26.6 ± 6.7	BMI: 24.9 ± 4.62	5.89 ± 1.95	Self: 4.39 ± 1.35 Typical female: 4.31 ± 1.47 Most attractive: 3.66 ± 1.19	BAS: + 1.64 ± 0.95; FID: 1.50 ± 1.06	Photographic Figure Rating Scale (10 photographic silhouettes; Swami et al. [56]; BAS: Body Appreciation Scale [57]; BMI (self-reported)
Swami et al. (2012) [25]	Zimbabwe (Harare)		Female: 140	18–46 Mean: 25.3 ± 6.87	BMI: 24.81 ± 4.61	4.54 ± 1.91	Self: 4.99 ± 1.10 Typical female: 4.71 ± 1.16 Most attractive: 5.17 ± 1.07	BAS: 1.19 ± 0.93; FID: −0.45 ± 0.31	Photographic Figure Rating Scale (10 photographic silhouettes; Swami et al. [56]; BAS: Body Appreciation Scale [57]; BMI (self-reported)
Tovée et al. (2006) [26]	South Africa (Mshwati Mpolveni)/Zulus	UK	Male: 25; Female: 27	Mean: 26.6 ± 6.87			Female ideal BMI: 23.99		50 high-resolution photographic images (self-administered questionnaire)
Tovée et al. (2006) [26]	Britons of African descent	UK/Second generation immigrants	Male: 34; Female: 32	Mean: 24.4 ± 4.53			Female ideal BMI: 20.68		50 high-resolution photographic images (self-administered questionnaire)
Tovée et al. (2006) [26]	South Africa (Mshwati Mpolveni)/Zulus		Male: 19; Female: 16	Mean: 25.6 ± 4.47			Female ideal BMI: 26.52		50 high-resolution photographic images (self-administered questionnaire)
McHiza et al. (2011) [34]	South Africa (Cape Town, urban area)		Female: 44	Mean: 38.5 ± 9.0	BMI: 32.1 ± 7.1	5.5 ± 1.9	4.4 ± 1.2	FID: 1.1 ± +2.0	8 silhouettes developed by Stunkard et al. [53]
Swami et al. (2010) [2]	South Africa (Cape Town, urban area)		Male: 52; Female: 48	Mean: 38.4 ± 11.1	BMI: 23.3 ± 3.8	Female Actual: 4.5	Female Ideal: 3.2		9 silhouettes developed by Thompson and Grey [50]; BMI (self-reported)
Swami et al. (2010) [2]	South Africa (KwaZulu-Natal, rural area)		Male: 45; Female: 60	Mean: 38.4 ± 11.1	BMI: 40.1 ± 10.4	Female Actual: 6.0	Female Ideal: 5.6		9 silhouettes developed by Thompson and Grey [50]; BMI (self-reported)
Jaeger et al. (2002) [33]	South Africa (Cape Town)/black origin		Male and Female: 21	19–23 Mean: 19.3 ± 0.9	BMI: 23.9 ± 4.3	4.3	2.6	FID: +1.7	10 silhouettes (self-administered questionnaire)
Peltezer et al. (2012) [35]	South Africa		Male: 100	≥18	BMI: 21.1			BASS: 3.95 ± 0.70	The Multidimensional Body-Self Relations Questionnaire [58]; BASS: Body-Areas Satisfaction Scale.
			Female:189	≥18	BMI: 23			BASS: 3.91 ± 0.73	The Multidimensional Body-Self Relations Questionnaire [58]; BASS: Body-Areas Satisfaction Scale.
Puoane et al. (2005) [36]	South Africa (Khayelitsha, Cape Town)/black origin		Female: 44	28–60 Mean: 43.2 ± 7.2	BMI: 40.0 ± 8.1 Uw: 0 % Nw: 4.7 % Ow: 4.7 % Ob: 90.7 %	Self-perception: Uw: 7 % Nw: 48 % Ow/Ob: 45 %	Preferred BMI: 27		8 silhouettes developed by Stunkard et al. [53]
Faber et al. (2005) [37]	South Africa (KwaZulu Natal, rural area)/black origin		Female: 187	25–55	Uw: 0 % Nw: 28.9 % Ow: 41.2 % Ob: 29.9 %			Sat: 37 % Dis: 11 % Lose weight: 8 % Ow-ob want to lose weight: 25 %%	Self-administered questionnaire
Senekal et al. (2001) [38]	South Africa (rural and urban area)/black origin		Female: 180	Mean: 20 ± 4.4	BMI: 22.6 ± 3.8 Uw: 25.7 % Nw: 52.5 % Ow: 16.8 % Ob: 5.0 %	Self-perception: Uw: 6.1 % Nw: 67.0 % Ow: 26.3 % Ob: 0.6 %			Body Shape Questionnaire by Cooper et al. [52]

### Studies on North Africans

Nicolau et al. [22] focused on body size preference and body size perception of Moroccan immigrants in the Netherlands (Amsterdam), reporting that most Moroccan women wished to be thinner than they were (Table 1). The majority of Moroccan men were unaware of being overweight. Another two studies [18, 23] found that both female Moroccan immigrants in the Netherlands and residents in Morocco expressed a preference for thin and normal body size. Many of them wished to lose weight.

Research on NA immigrant women in Italy compared to Moroccan and Tunisian residents in their countries [24] suggested that there were great similarities in the ideal figure among NA immigrant women and Tunisian residents while Moroccan residents tended to prefer a heavier ideal figure. Body image dissatisfaction was slightly (albeit not significantly) higher in NA immigrants (FID: 1.99 ± 2.31) than in NA residents (Moroccans FID: 1.50 ± 1.83; Tunisians FID: 1.40 ± 2.41).

As regards NA residents, misperception of body weight [30] and a preference for fat body size [27, 28] were reported in three studies on Moroccan populations (Table 1). In particular, despite their high percentage of normal weight and overweight, Amazigh men and women from the High Moroccan Atlas underestimated their body mass and wished to gain weight [30]. Moroccan Saharoui women [27, 28] were generally satisfied with their body weight (almost 80 % satisfaction) in spite of the high prevalence of overweight and obesity, with a small percentage of them wishing to gain weight and an even lower percentage wishing to lose weight. Moroccan women living in Casablanca [29] had a high percentage of overweight and obesity, but most of them were not aware of their body size: about half of the normal weight women considered themselves too thin, while most of the overweight and obese women considered themselves “normal weight”. Another study taking in consideration Moroccan women living in Casablanca [24] reported that they were aware of their body size but they are dissatisfied because they wanted to be thinner. Also Egyptian university students were dissatisfied with their body weight, especially women [31, 32], which is consistent with the results obtained by Gualdi et al. [24] and by Nicolau et al. [23] on NA residents. In Tunisia, Tlili et al. [17] reported that over half of the women in a peri-urban area of Tunis were dissatisfied with their body weight, with most of them preferring a lighter ideal body size and a smaller proportion wanting a heavier one. Moreover, the majority of overweight and obese women preferred a slimmer silhouette. A normal body size was generally seen in the most positive light, although some positive attributes were associated with overweight. An association between BMI or body size preferences and age or level of education was found in this study [17]. Consistently with these results, the young Tunisian men and women examined by Jaeger et al. [33] and Tunisian women examined by Gualdi-Russo et al. [24] were aware of their body size (normal weight or slightly overweight), but they wished to be thinner.

### Studies on Central Africans

As already mentioned, no studies were found regarding CA immigrants in EU. However, in order to have a complete picture of body image perception and beauty ideals in Africa, we included five studies on CA residents from different countries in Table 1. Three of them considered Ghanaian women, reporting their preference for a slightly overweight or normal weight body shape and rather low level of FID [4, 33, 39]. The studies of Frederick [4] and Benkeser et al. [39] found that, unlike normal weight Ghanaian men and women studied by Jaeger et al. [33], there was a misperception of their weight status since, despite their obesity status (according to BMI), they saw themselves as overweight [4, 39]. Siervo et al. [40] reported that Gambian men and women had a preference for overweight body shapes. In particular, the oldest group of men and women wanted to be fatter. Higher body dissatisfaction emerged only in the oldest group of females, since they were aware of being obese and wanted to be slightly slimmer. Senegalese women generally had a good perception of their body [41], showing only slight body dissatisfaction because their ideal was a little slimmer than their actual figure (slightly overweight). Both male and female residents in Nigeria and Gabon had, on average, low levels of FID and a normal BMI. They had good perception of their body and a preference for normal body size [33, 42]. Moreover, the majority of Nairobi slum residents were in normal weight with a tendency for men to overestimate their body size and for women to underestimate it. Unlike men, women had low levels of dissatisfaction with a preference for normal body size [43].

In general, CA residents showed a preference for normal or slightly overweight body size, with a rather low level of dissatisfaction with their body.

### Studies on Southern Africans

Two studies of SA migrants in EU reported the results for Zulus and Zimbabweans who had migrated to the UK [25, 26]. Both groups of immigrants wanted to lose weight and had general preferences for normal weight body size. The female Zimbabwean immigrants in the UK [25] had a more negative body image perception than their peers in Zimbabwe and their level of dissatisfaction was very high. They reported a greater weight discrepancy because they saw themselves as larger than they were to a greater degree than the Zimbabwean residents and they wanted a thinner body size. Instead the Zimbabwean women living in Zimbabwe still held to an ancestral idea of beauty, preferring a heavier body [25]. In Tovée’s study [26], there were substantial differences in the perception of female attractiveness in the different groups of immigrants examined. The peak of attractiveness for Zulus resident in South Africa shifted to a higher BMI, while for Zulus who moved to Britain (Female ideal BMI: 23.99) it seemed to be intermediate between those of their peers living in South Africa and Britons of African descent.

Five studies reported results for SA residents in urban (Cape Town) and rural (KwaZulu-Natal) areas [2, 33, 34, 36, 37]. Both rural and urban groups wished to lose weight but the greatest body discrepancy was among the urban residents, who preferred normal weight figures in contrast to the rural inhabitants who preferred an overweight body shape. Additionally, other two studies [35–38] found the presence of body dissatisfaction among South African residents in urban and rural areas, with normal weight men and women slightly overestimating their body weight.

### Summary and general geographical pattern

In order to clarify the complex picture emerging from the literature, this paragraph provides a summary (Table 2) of the main results examined in detail above. It is organized around the main aims of this study focusing on weight status and body image perception in African residents and immigrants, compared to European populations, when possible.

**Table 2:** Summary of the main results from literature according to the specific aims of this study

Body image and weight perception: general tendency and differences among samples living in the same area or country	North Africa
	• General misperception of body weight and preference for normal weight or slightly overweight body silhouettes [17, 23, 24, 27–33] • Underestimation of body weight and preference for overweigh/obesity in rural populations [27, 28, 30]; preference for thinner body [24, 33] and dissatisfaction [24, 33] in urban or peri-urban populations.
	Central Africa
	• General preference for normal- slightly overweight body size and a rather low level of dissatisfaction with their body in rural and urban populations [4, 33, 39–43]; • Gambia: preference for overweight among oldest people and for slightly slimmer body among younger. Older Gambians were the most dissatisfied [40]. • Senegal: preference for a little slimmer figures than actual ones [41]. • Ghana: low level of dissatisfaction and preference for normal weight or overweight body size both in rural and urban area [4, 33, 39]
	Southern Africa
	• Preference for normal weight figures and great body discrepancy in urban residents [2, 33, 34]; preference for overweight body shape in rural inhabitants [2, 25, 26].
Body image and weight perception: differences between women and men according to area and country	North Africa
	• Morocco: weight underestimation in both Amazigh men and women (High Moroccan Atlas) and wish to gain weight [30]; • Egypt: higher level of satisfaction and less body image concern in male students compared to females [31, 32].
	Central Africa
	• Gambia: less dissatisfaction and preference for bigger body shape in men than in women [40]; • Nigeria: similar preferences in body shape among men and women, but higher dissatisfaction in men [42]; • Kenya: overestimation of body size in normal weight men and underestimation in women living in slums in Nairobi. Unlike men, women had low levels of dissatisfaction with a preference for normal body size [43].
	Southern Africa
	• South Africa: similar level of satisfaction in males and females [35].
Interaction between weight status and body image perception according to area and country	North Africa
	• Morocco: underestimation of body weight and consequent preference for fatter body [25, 27, 28, 30]. • Tunisia: awareness of body weight and preference for a thinner body [17, 24, 33]. • Egypt: good awareness of their body weight and high level of dissatisfaction [31, 32]
	Central Africa
	• Ghana: misperception of weight status (obesity) in women [33, 39]. • Nigeria and Gabon: low levels of FID and a normal BMI. Residents had good perception of their body and a preference for normal body size [33, 42] • Kenya: residents of Nairobi slums were, on average, in normal weight with a differently-oriented misperception in men and women [43].
	Southern Africa
	• South Africa: low level of body dissatisfaction in normal weight men and overestimation of body weight in women [35].
Comparison in body image and weight perception of African immigrants in Europe with residents in the original country	North Africa
	• Moroccan female immigrants in the Netherlands: preference for thin and normal body size such as among the female residents in Morocco urban area. Many of them wished to lose weight [18, 22, 23]. • NA immigrant women in Italy: preference for thin silhouettes, as among Tunisian residents. The ideal silhouette was slightly heavier in Moroccan residents than in Tunisians and immigrants. Body image dissatisfaction was slightly higher in NA immigrants than in NA residents [24].
	Central Africa
	• No studies on CA immigrants in Europe are available.
	Southern Africa
	• Zimbabwean immigrants in the UK: they were highly dissatisfied and with a more negative body image perception than residents in Zimbabwe, with a preference for a thinner body size while women living in Zimbabwe preferred a heavier body [25]

**Fig. 2:**
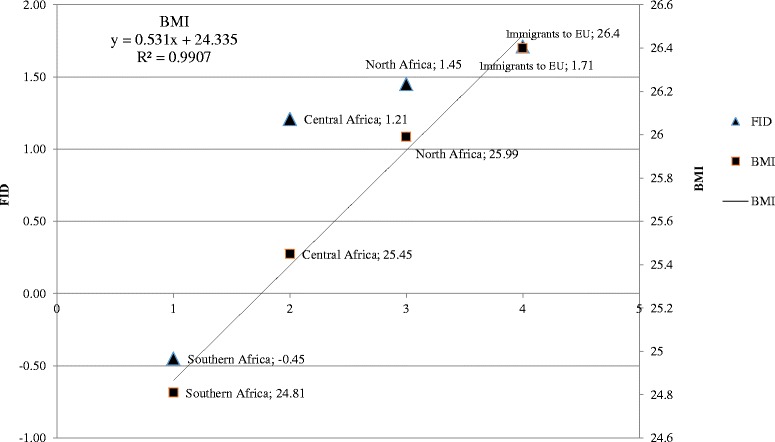
Geographical gradients in FID and BMI from Southern Africa to North Africa and EU

To acquire a synthetic overview of body image perception in people with an African origin, we considered resident and immigrant African samples, combining the data from several studies for the following geographical areas: Southern Africa, Central Africa, North Africa, EU (immigrants from Africa). This analysis was possible only for females (Southern Africa: 140 Zimbabwes from Swami et al., 2012 [25]; Central Africa: two Gambian samples differently aged, 50 subjects each, from Siervo et al. [40]; North Africa: 124 Moroccans and 104 Tunisians from Gualdi-Russo et al. [24]; EU: 138 Zimbabwe immigrants to UK from Swami et al. [25] and 105 NA immigrants to Italy from Gualdi-Russo et al. [24]. Figure 2 shows the linear relationship between BMI and the geographical areas (statistically significant). The FID also increased significantly with the geographical gradient (Spearman R = 1). The mean increase in BMI was 0.53 kg/m^2^ per unit increase in geographical area (Fig. 2).

**Fig. 3:**
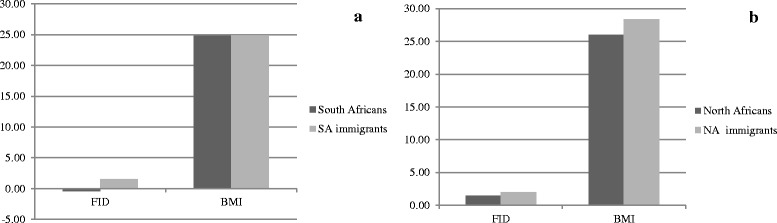
Comparisons between immigrants in EU and African residents for FID and BMI. Graphs show the differences between the mean values of subjects with SA origin (**a**) or NA origin (**b**)

A different trend emerged for North Africa and Southern Africa (Fig. 3) when immigrants were compared with residents in the country of origin: SA immigrant women had higher values of FID than SA residents, but similar BMI values; NA immigrant women had significantly higher values of BMI (p < 0.05) than NA residents, but similar values of FID.

## Discussion

In many non-Western and low-income countries, body fat is seen as an indicator of health and prosperity since only high-status individuals have the possibility to put on body weight because of the scarcity of food. Conversely in many industrialized countries with a food abundance, fatness is associated with poverty and poor health [17] while slimness is a sign of high economic status [44]. A recent study of South Africans [45] revealed that overweight women underestimate their size and have an inappropriate perception of the risk of the obesity. However, it was suggested that the degree of preference for plumpness in non-Western societies has been exaggerated by Western studies and that the fear of fatness is more likely to be expressed in those who have had a greater exposure to Western culture [46].

During the complex immigration process, everything that surrounds the person changes, including the diet, social and family relationships, climate and culture [47]. Factors such as acculturation, enculturation and socio-economic status (SES) have a strong effect on many parameters, including weight status and perception of body size.

The data analysis in the present review revealed a wide variability of body weight/image perception in Africa: African residents generally showed a preference for heavier body size than their immigrant peers, but differences in preferences were evident in relation to the area of residence, ethnicity, and social and cultural factors even in the same population (i.e. Moroccans). Similarly, in a longitudinal study on changes in body composition of black urbanised South African women [48], the presence of two different groups of overweight/obese women was highlighted: one group, more aligned with Western values, wished to be thinner and the other one, more aligned with the African values, was content with its body size.

Some of the considered ethnic groups desired a thinner body image while others held to the idea that higher body weight is a positive factor. This puts the latter groups at higher risk of obesity and the maintenance of it over time. A high level of body size dissatisfaction can lead to eating disorders and poor eating habits.

Hence this review shows that there is a different awareness of weight among the examined groups, with different consequences for health and well-being.

We found in the analysis of general geographical pattern that the body image dissatisfaction (FID) increased with the increase in BMI from south to north, reaching the highest values in African immigrants in EU, who also had the highest values of BMI (on average, above the cut-off of overweight). These results indicate different beauty ideals of African populations and thus different degrees of satisfaction in the perception of their body image from Southern Africa to North Africa and, to a greater degree, with the migration to Europe. In addition to differences in ideals, there were changes in weight status, which increased from Southern Africa to North Africa and to EU.

The differences, for example, between Zulu immigrants and those resident in South Africa were explained in terms of adaptation to a new environment [26]. The adaptation to the new environment was supported by the comparison between UK Africans and UK Caucasians (BMI: 20.85) since both preferred medium BMI values. The beauty ideals of the immigrants became similar to those of the host Western population and increased the level of dissatisfaction, placing them at greater risk for the development of a negative body image and eating disorders [25].

There are some limitations to this review. Our synthesis of the African trend according to a geographical gradient is limited by the uncertainty involved in the data used to calculate the mean values of FID and BMI for each area. This uncertainty relates to the fact that only a few countries and small samples were available for each area. Another limitation of the review is the variety of methods utilized to assess body image and body size preferences, so that a comparison between different studies is difficult. Many authors did not utilize appropriate silhouettes for their studies or used self-administered questionnaires without figural references. Some studies were based on self-reported height and weight and this could result in an incorrect estimate of the BMI. Moreover, the studies did not report the same information, often overlooking one or more data (i.e. actual self-perception or the ideal body size). Nevertheless, due to the scarcity of studies focusing on body image perception and body size ideals in immigrants in Europe, we considered necessary to include all the studies reported in the literature in this review. Finally, only a few studies considered individuals of both sexes and the majority of them focused on female body shape or did not separate the results between the two sexes.

## Conclusions

Although there are still few specific studies reported in the literature, our review is a good starting point to evaluate body image perception and body ideals in populations of African origin. The results for African immigrants in Europe suggest that body dissatisfaction is increasing with respect to populations living in the country of origin. There is a tendency to a simultaneous increase in BMI and a decrease in FID from Southern Africa to North Africa and Europe.

Therefore, the results highlighted in this review are evidence of preferences for slimness during the process of acculturation (for immigrants) and, more in general, of Westernization, involving greater body dissatisfaction and the risk of nutritional disorders.

The availability of local data on body image perception is essential to plan health strategies and to support non-communicable disease management. Continued monitoring of how the perception of body image is evolving, with a check of the changes of weight ideals over time, should be a primary health goal to ensure psychological well-being and health among immigrants in Europe.

## Abbreviations

BMI, body mass index; CA, Central African; DIS, dissatisfied; EU, Europe; FID, feel minus ideal difference index; NA, North African; NW, normal-weight; OB, Obese; OW, Overweight; SA, Southern African; SAT, Satisfied; SD, Standard deviation; SES, Socio-economic status; UW, Underweight
